# Super-twisting sliding mode control of dopamine release in VTA dopaminergic neuron model: a simulation study

**DOI:** 10.1038/s41598-026-54776-7

**Published:** 2026-05-28

**Authors:** Najme Soheilipour, Amir Akhavan, Ehsan Rouhani, Raha Rahimi

**Affiliations:** Department of Electrical and Computer Engineering, Isfahan University of Technology, Isfahan, 84156-83111 Iran

**Keywords:** Super-twisting, Sliding mode control, Dopamine, Ventral tegmental area, Firing rate, Engineering, Neuroscience

## Abstract

Modulation of dopaminergic signaling has emerged as a central topic in cognitive neuroscience, particularly in relation to neurological and psychiatric disorders. Although numerous computational models have been developed to investigate dopamine-related dynamics, most existing regulation approaches rely on open-loop stimulation strategies or biologically descriptive formulations. Such approaches lack feedback mechanisms and therefore cannot systematically compensate for parameter uncertainties, physiological variability, or external disturbances, which limits their robustness and practical applicability. To address these limitations, this study proposes a robust closed-loop control framework based on super-twisting sliding mode control (ST-SMC) for regulating dopamine release in a microscopic ventral tegmental area (VTA) dopaminergic neuron model. The controller is designed to ensure robustness against significant parameter uncertainties (up to 75%) and external disturbances without requiring explicit system identification. Regulation is implemented at two complementary levels: direct membrane voltage control and firing rate–based control derived from membrane voltage dynamics. The firing rate formulation provides a practically measurable and clinically relevant control objective, particularly when direct membrane voltage regulation is not feasible. Simulation results demonstrate superior performance of the proposed method compared with proportional (P), proportional–integral–derivative (PID), and dual-threshold (DT) controllers. For membrane voltage regulation, the ST-SMC achieves a mean RMSE of 0.31 mV, outperforming P (0.65 mV), DT (17.57 mV), and PID (1.43 mV) controllers. For firing rate control, the proposed approach attains a mean RMSE of 0.67 Hz, compared to 0.94 Hz, 2.06 Hz, and 3.00 Hz for P, DT, and PID, respectively. Although the control energy in the firing rate scenario is slightly higher than that of the P controller, the method provides substantially improved tracking accuracy, reflecting an effective trade-off between precision and stimulation effort. Overall, the proposed ST-SMC framework offers a robust, accurate, and computationally efficient solution for closed-loop regulation of dopaminergic activity.

**Supplementary Information:**

The online version contains supplementary material available at 10.1038/s41598-026-54776-7.

## Introduction

The ventral tegmental area (VTA), located in the midbrain, is a major dopaminergic nucleus and one of the brain’s primary centers for dopamine secretion. It plays a pivotal role in regulating a wide range of behavioral and cognitive functions, including reward processing, motivation, aversion, stress modulation, learning, memory, and addiction. Approximately 60% of VTA neurons are dopaminergic, projecting through key pathways such as the mesolimbic and mesocortical systems^1,2^. The mesolimbic pathway transmits dopamine to regions like the nucleus accumbens and amygdala, mediating reward-related behavior and emotional responses, while the mesocortical pathway connects to the prefrontal cortex, influencing executive functions and decision-making. Dopamine, the central neurotransmitter in these circuits, modulates complex behaviors and is essential for maintaining motor function, memory, and emotional regulation^3^. Disruptions in dopaminergic signaling particularly within the VTA have been implicated in a range of neurological and psychiatric disorders, including Parkinson’s disease^4^, Alzheimer’s disease^5^, schizophrenia^6^, attention-deficit/hyperactivity disorder (ADHD)^7^, mood disorders^8^, and drug addiction disorders^9^. Given the increasing societal burden of reward-related disorders, a comprehensive understanding of the VTA and its role in dopamine modulation is critical^10–13^. Recent advances in computational neuroscience have emphasized dopamine modeling as a powerful tool to decipher the dynamics of the reward circuit. Such models allow for the simulation of dopaminergic activity and its behavioral consequences, offering valuable insights into disease mechanisms. In particular, modeling dopamine release in the VTA has emerged as a critical step toward understanding the neurobiological basis of psychiatric and neurological disorders. By capturing the complex interplay of cellular and synaptic processes governing dopaminergic signaling, these models provide a framework to investigate how disruptions in dopamine dynamics may lead to dysfunctions observed in conditions such as addiction, depression, and schizophrenia. Ultimately, such computational approaches pave the way for more precise diagnostics, early interventions, and the development of targeted therapeutic strategies, offering a promising path toward the treatment of cognitive and affective disorders^14^.

Several computational models of the reward circuitry have been proposed to enhance understanding of dopamine release mechanisms from the VTA^15–19^. Wiencke et al.^19^ introduced a computational model of dopamine transmission, simultaneously considering dopamine release, diffusion, and uptake in both synaptic and volume transmission contexts, to simulate dopamine concentration variability. Yin et al.^15^ developed a biophysical model of the prefrontal cortex to explore how dopamine affects neuronal activity and compulsive behaviors in obsessive-compulsive disorder (OCD), suggesting that high dopamine levels can induce persistent network activity leading to obsessive tendencies. Shumilov and Gotovtsev^16^ modeled the dopamine signaling pathway using a combination of analog electrical circuits and mathematical approaches. By integrating cytomorphic electronic circuits with mathematical models, they simplified calculations and demonstrated the dynamics of the signaling pathway. Their model examines the effects of changes in calcium channel function and offers insights into dopamine-related cognitive diseases such as Parkinson’s disease. Đorđević et al.^17^ introduced a nonlinear stochastic model describing dopamine synthesis, storage, release, uptake, and metabolism in the rat striatum. Their model incorporates a 9-dimensional Brownian motion to capture the inherent randomness in dopamine dynamics. The authors prove the existence and uniqueness of a positive global solution, providing bounds for specific moments of coordinate processes. By simulating the model using a positivity-preserving balanced implicit method, they illustrate theoretical results, offering insights into dopamine regulation. Li et al.^18^ developed a computational model of the VTA-NAc-mPFC neural circuit to investigate the impact of dopamine concentrations on major depressive disorder (MDD). Using the Hodgkin-Huxley model and a dynamic receptor binding model, they simulated how varying dopamine levels affect neural activity patterns. Their findings suggest that reduced dopamine concentrations in MDD can disrupt the excitation-inhibition balance within this circuit, leading to pathological patterns observed in the disorder. This model offers a quantitative framework for understanding the role of dopamine in MDD and for identifying potential drug targets. Having reviewed various models of the brain’s reward circuitry, recent research trends have increasingly focused on modeling the VTA, given its central role in regulating dopamine release. This shift reflects a growing recognition that precise control over dopaminergic activity in the VTA may offer significant benefits in understanding and treating cognitive disorders. Indeed, modulation of dopaminergic signaling has emerged as a central theme in cognitive neuroscience, especially in the context of neurological and psychiatric conditions. In contrast, closed-loop control systems continuously monitor the output and adjust the control input accordingly, enabling automatic compensation for disturbances and parameter changes. As such feedback-based control strategies’s therapeutic potential for targeting dopaminergic pathways becomes more evident, the development of robust frameworks for manipulating dopamine release is gaining momentum, laying the groundwork for innovative control-based interventions with far-reaching clinical applications. Despite these valuable contributions, the majority of existing dopamine-related studies primarily focus on biological interpretation, phenomenological modeling, or explanatory analysis of specific neurobiological, biochemical, or circuit-level mechanisms. Consequently, the current literature often provides fragmented descriptions of dopaminergic activity and offers relatively limited unified nonlinear dynamical formulations that are explicitly suited to support regulation-oriented approaches for modulating dopamine release under external intervention. This highlights an open research gap in developing modeling frameworks that bridge physiologically grounded neuronal dynamics with control-oriented tractability. In this study, the employed VTA model is a hybrid and simplified conductance-based formulation adapted from established biophysical models, integrating membrane voltage dynamics, population-level GABAergic inhibition, and spike-triggered dopamine release. The model is deliberately structured to balance physiological realism with suitability for systematic closed-loop control design and evaluation.

A number of open-loop and closed-loop approaches have been introduced to control dopamine dynamics, typically through predefined intake patterns or pharmacological simulations. Chou and D’Orsogna^20^ developed an open-loop control approach through a dynamical systems model of drug addiction, where dopamine-related responses evolve based on user-driven intake patterns. Their model simulates addiction by recursively adjusting physiological parameters specifically those mediating the balance between pleasurable and aversive states. Control is exerted via feedback from reward prediction error (RPE), influencing drug dose and frequency. This structure allows investigation into personalized addiction trajectories and treatments like methadone. However, many existing approaches for dopamine modulation rely on open-loop stimulation strategies, in which control or stimulation signals are predefined and applied without feedback from the system. In such formulations, the stimulation profile remains fixed during operation and does not explicitly account for ongoing changes in neuronal activity. In biological contexts such as dopamine-regulated 3,4-dihydroxyphenylalanine (DOPA) homeostasis, these open-loop strategies are typically implemented using predefined stimulation patterns, meaning that variations or perturbations cannot be accommodated through real-time feedback and often require manual adjustment of stimulation parameters. As a result, open-loop approaches are primarily suitable for exploratory analysis or predefined experimental conditions, rather than for systematic regulation of dopaminergic dynamics. In contrast, closed-loop control systems continuously monitor the output and adjust the control input accordingly, enabling automatic compensation for disturbances and parameter changes. Such feedback-based control strategies have also been explored in biomedical applications, particularly in the context of automated therapeutic regulation, highlighting the practical relevance of closed-loop control in medical systems^21,22^. Kleppe et al.^23^ employed a closed-loop control system to model dopamine -mediated homeostasis of DOPA in presynaptic neurons. Using a control-theoretic and computational approach, they show that dopamine acts as a derepression regulator via a negative feedback loop with tyrosine hydroxylase (TH), dynamically adjusting DOPA synthesis to maintain stability under perturbations like oxidative stress or Levodopa treatment. The model incorporates zero-order kinetics for vesicular dopamine transport and robust integral control, ensuring DOPA levels remain homeostatic despite enzymatic or environmental changes. Their findings highlight dopamine’s dual role in stabilizing DOPA and preventing pathological dopamine accumulation, with implications for neurodegenerative and addiction-related disorders. However, a closer examination of the existing literature indicates that dopamine-related modeling and modulation studies predominantly rely on open-loop stimulation strategies or predefined input patterns, with limited emphasis on systematic control-oriented formulations. Although a small number of works adopt feedback-based formulations, their scope is generally limited to biochemical regulation mechanisms rather than the explicit control of electrophysiological neuronal dynamics at the microscopic VTA level. Consequently, direct and fair comparison with control-oriented closed-loop frameworks tailored to microscopic VTA neuron models remains limited in the current literature.

To overcome the aforementioned limitations of open-loop dopamine modulation strategies, and to further motivate the development of an engineered closed-loop control framework for regulating dopamine release dynamics within a microscopic VTA neuron model, in this study, a robust closed-loop control algorithm based on the super-twisting sliding mode control (ST-SMC) approach is proposed. In this work, ST-SMC is adopted as a benchmark robust control tool and is formulated for the considered microscopic VTA neurodynamic setting, with the main contribution lying in the control-oriented problem formulation and regulation objectives rather than in modifying the underlying ST-SMC theory. The robustness and effectiveness of the ST-SMC framework have been validated in a variety of complex and nonlinear systems across different application domains^24–26^. While previous closed-loop studies, such as the work of Kleppe et al.^23^, primarily focus on intrinsic biochemical feedback and homeostatic regulation of dopamine precursors within a systems biology framework, they do not explicitly address the synthesis of a robust control law designed to regulate electrophysiological neuronal dynamics under uncertainties and disturbances. The proposed ST-SMC controller is specifically designed to withstand uncertainties in system parameters as well as external disturbances, ensuring reliable performance under various physiological and pathological conditions. Moreover, in comparison with advanced PID-based feedback controllers including neural, bio-inspired, and fractional-order variants, the ST-SMC framework provides finite-time convergence, inherent robustness to bounded uncertainties and disturbances, and a fully control-oriented design supported by rigorous stability analysis, without relying on tuning- or learning-based mechanisms. These properties make ST-SMC particularly suitable for nonlinear and uncertain dopamine neurodynamic systems. Additionally, unlike adaptive or learning-based PID variants, the proposed ST-SMC preserves its performance using a fixed set of controller parameters across both nominal operating conditions and scenarios involving significant uncertainties and disturbances, without requiring retuning or controller reconfiguration. The effectiveness of the proposed control strategy is evaluated using two complementary modeling approaches based on membrane voltage dynamics and neuronal firing rate of the VTA model. Notably, controlling the neuronal firing rate is more practical than directly manipulating membrane voltage, as it serves as a stable, high-level feature derived from the underlying voltage dynamic. The performance of the proposed controller is compared with proportional (P), proportional–integral–derivative (PID)-based, and dual-threshold (DT) control schemes, which are commonly used as baseline feedback controllers in practical neuromodulation and biomedical control applications. These controllers are selected due to their simple structure, low computational complexity, and proven feasibility in experimental settings, making them particularly compatible with firing rate–based regulation frameworks, where robustness, interpretability, and ease of implementation are of primary importance. The results demonstrate that the proposed STMSC achieves higher accuracy in the presence of disturbances and model uncertainties, outperforming both baseline controllers and offering a comprehensive framework for regulating the dopaminergic activity through advanced control mechanisms. The main contributions of this study can be summarized as follows:

- A control-oriented nonlinear dynamical formulation of a microscopic VTA neuron model is adopted and extended by incorporating a finite inhibitory GABAergic neuronal population, enabling systematic regulation of dopamine-related neural dynamics beyond purely descriptive or phenomenological modeling.
- A robust closed-loop control strategy based on the ST-SMC framework is developed for regulating dopamine-related neural activity, explicitly addressing system nonlinearities and uncertainties without relying on adaptive tuning or learning-based mechanisms, and its effectiveness is validated through comprehensive simulations under different operating conditions.
- The proposed control framework is implemented at two complementary regulation levels by enabling both direct membrane voltage regulation and firing rate-based regulation derived from membrane voltage dynamics, with the latter providing a higher-level and practically meaningful control objective when direct membrane voltage measurement or regulation is not feasible.

The paper is organized as follows. The dynamic modeling of the VTA neuronal population, along with the subsequent design of ST-SMC, is comprehensively described in section "Methods". Section "Results" covers the simulation outcomes, which are discussed in section "Discussion". The study concludes with the findings in section "Conclusion".

## Methods

**Fig. 1:**
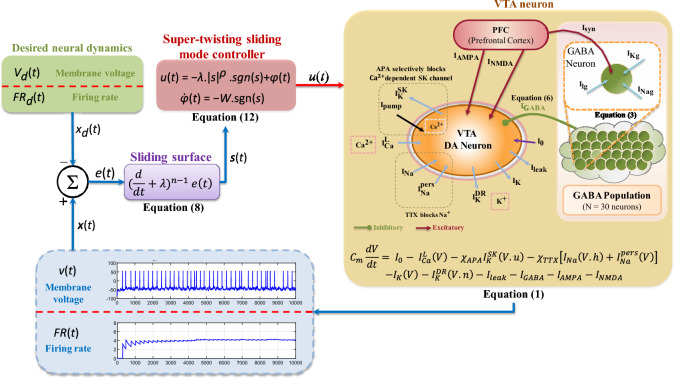
Block diagram of the closed-loop control system incorporating the DA neuron and a population of GABAergic neurons. The output of the plant is the membrane voltage or the firing rate of the DA neuron.

In this section, the dynamic model of the VTA population is presented. The model incorporates the primary neuronal component of the VTA, a dopaminergic (DA) neuron, and a population of inhibitory GABAergic neurons affected by DA neuron activity. Figure 1 illustrates the whole structure of the VTA model serving as the plant for subsequent controller. The membrane voltage and the firing rate of the DA neuron, as key neuronal characteristics, are considered as the outputs of the VTA neuron model to design the control method.

### VTA dopaminergic neuron model

To construct a modeling framework for the VTA DA neuron that facilitates the introduction of a control input in the form of an electric current, the established conductance-based dynamic model of the DA neuron originally presented by Oster et al.^27^, is employed. This model examines the voltage activity of the neuronal membrane at a microscopic level, accounting for principal intrinsic and synaptic currents of the neuron. The key advantage of this model is its capacity to apply a control input as an electric current to the neuron. The biophysical model of the DA neuron is described as a single-compartment conductance-based model as follows:

1 $$\begin{aligned} C_{m} \frac{{dV}}{{dt}} & = I_{0} - I_{{Ca}}^{L} \left( V \right) - \chi _{{APA}} I_{K}^{{SK}} \left( {V.u} \right) - \chi _{{TTX}} \left[ {I_{{Na}} \left( {V.h} \right) + I_{{Na}}^{{pers}} \left( V \right)} \right] \\ & - I_{K} \left( V \right) - I_{K}^{{DR}} \left( {V.n} \right) - I_{{leak}} - I_{{GABA}} - I_{{AMPA}} - I_{{NMDA}} \\ \end{aligned}$$

where, *V* represents the membrane voltage, and ${C}_{m}$ (1 $\mu F/{cm}^{2}$) is the membrane capacitance of the DA neuron. ${I}_{0}$ is an external current or the general unmodeled in vivo input currents to the DA neuron. The intrinsic currents of DA neuron are L-type calcium current (${I}_{Ca}^{L}$), an apamin sensitive, calcium dependent SK-type potassium current (${I}_{K}^{SK}$), spike-generating sodium current (${I}_{Na}$), persistent sodium current (${I}_{Na}^{pers}$), generic potassium current (${I}_{K}$), delayed-rectifier potassium current (${I}_{K}^{DR}$), and leakage current (${I}_{leak}$). The currents in the model can be categorized into three categories. The first subgroup of intrinsic currents, comprising ${I}_{Na}$, ${I}_{K}^{DR}$, ${I}_{K}$, ${I}_{Na}^{pers}$, and ${I}_{leak}$, are voltage-dependent and responsible for spike generating. The administration of apamin selectively blocks the SK current (${I}_{K}^{SK}$), which is primarily dependent on voltage and cytosolic calcium concentration, denoted by *u*. Increasing apamin concentration progressively blocks SK channels, thereby enhancing the influence of ${I}_{Ca}^{L}$ on subthreshold membrane voltage dynamics. The parameter ${\chi}_{APA}$ is correlated with the efficacy of the SK channels, where ${\chi}_{APA}=1$ signifies no apamin application, and ${\chi}_{APA}=0$ indicates complete blockage of SK channels due to high apamin concentration. Tetrodotoxin (TTX) blocks the sodium current, which is essential for spike generating. When TTX is applied (${\chi}_{TTX}=0$) the model reveals underlying subthreshold membrane voltage fluctuations^28^. The second subgroup of intrinsic currents includes calcium and calcium-dependent currents (${I}_{Ca}^{L}$, ${I}_{K}^{SK}$). The third subgroup encompasses synaptic currents: α-amino-3-hydroxy-5-methyl-4-isoxazolepropionic acid (AMPA) and N-methyl-D-aspartate (NMDA) receptor currents (${I}_{AMPA}$ and ${I}_{NMDA}$, respectively). Synaptic inputs can trigger both burst firing and termination of spikes. Note that for the sake of simplicity, the currents with minimal effect on VTA pacemaking have been excluded from the model and incorporated into ${I}_{0}$. The detailed dynamics of the intrinsic and synaptic currents are provided in Appendix A of the Supplementary Materials. The dopamine release from a DA neuron is modeled through Equation (2), using the dopamine diffusion framework with the following equation^29^:

2 $$\frac{d\left[DA\right]}{dt} = {\left[DA\right]}_{max}\delta \left(t-{t}_{spike}\right)-\frac{{V}_{max}\left[DA\right]}{{K}_{m}+\left[DA\right]}$$

where, the Dirac delta function, $\delta \left(t-{t}_{spike}\right)$, captures the precise timing of dopamine release at the moment of a spike occurrence, with the maximum amount of dopamine released per spike, ${\left[DA\right]}_{max}=0.1 \mu M$, ${V}_{max}=0.004 \mu M/ms$ represents the maximum reuptake rate by a transporter, and ${K}_{m}=0.2 \mu M$ denotes the transporter’s affinity for dopamine.

### VTA GABAergic neuron model (${{\boldsymbol{I}}}_{{\boldsymbol{G}}{\boldsymbol{A}}{\boldsymbol{B}}{\boldsymbol{A}}}$)

GABAergic inhibition of DA neurons in the VTA is a critical factor modulating dopaminergic activity. To accurately represent this influence within a computational model, we incorporate a population of 30 GABAergic neurons, with the single-neuron dynamics following the model described by Morozova et al.^30^, projecting to a single dopamine neuron. This approach enhances the model’s physiological realism by capturing the complex interactions within the VTA so that a refined model of the GABAergic synaptic input to the DA neuron (${I}_{GABA}$ in Equation (1)) is represented (Equation (6)), incorporating the collective dynamics of the GABAergic population. The membrane voltage dynamics of each VTA GABAergic neuron are described by^30^

3 $${C}_{m}\frac{d{v}_{i}}{dt} = {I}_{Nag}+{I}_{Kg}+{I}_{lg}+{I}_{syn}$$

where, ${v}_{i}$ denotes the membrane voltage of the *i*-th GABA neuron within a population of GABAergic neurons and ${C}_{m}=1\mu F/{cm}^{2}$ is the membrane capacitance of the GABA neurons. ${I}_{Nag}$, ${I}_{Kg}$ and ${I}_{lg}$ represent the sodium, the potassium, and​​ the leak currents of the neurons, respectively. Additionally, ${I}_{syn}$​ corresponds to the synaptic currents received by GABA neurons. The intrinsic firing frequencies of these neurons are typically regulated within the range of 12 to 22 Hz. Synchronous or asynchronous activity of GABA neurons can affect the firing rate of DA neuron. Asynchronous activity of GABA neurons results in varying levels of inhibition for DA neurons. However, when these GABAergic neurons are synchronized by synaptic inputs (${I}_{syn}$), this synchronization elicits additional spike activity in dopamine neurons, resulting in an increased firing rate and a subsequent reduction in the inhibition of DA neurons activity^31^. In the current model, the inhibitory role of GABAergic population on dopamine was desired and therefore, the asynchronous activity of GABA neurons was considered in the model and the synaptic current (${I}_{syn}$) was omitted from the model. The dynamics of ${I}_{Nag}$, ${I}_{Kg}$ and ${I}_{lg}$ currents are detailed in Appendix B of the Supplementary Materials. The modulation of the gating variable ${s}_{{GABA}_{i}}$ of the *i*-th GABA neuron is described as follows^30^:

4 $$\frac{d{s}_{{GABA}_{i}}}{dt}=\frac{{g}_{spike}({v}_{i})(1-{s}_{{GABA}_{i}})}{{\tau}_{gact}}-\frac{(1-{g}_{spike}({v}_{i})){s}_{{GABA}_{i}}}{{\tau}_{gdeact}}$$

$$\text{where, } \tau_{gact} = \frac{1}{12} \, ms \text{ and } \tau_{gdeact} = 10 \, ms \text{ and } g_{spike}(v_i) \text{ is obtained from}$$

5 $${g}_{spike}\left({v}_{i}\right)=\frac{1}{1+\mathrm{e}\mathrm{x}\mathrm{p}(\frac{-{v}_{i}}{2})}$$

With each firing event, the GABAergic neurons release a specific quantity of neurotransmitter. In order to model the activation of the GABAergic current into the DA neuron, the aggregate contributions from all GABAergic neurons within a given population^31^, ${s}_{GABA}$ is calculated as the sum of the ${s}_{{GABA}_{i}}$ values for all GABAergic neurons, normalized by the total number of neurons to ensure its value lies between 0 and 1^30^. The GABAergic synaptic current from VTA GABAergic neurons to the DA neuron (${I}_{GABA}$ in Equation (1))^30^ is calculated with

6 $${I}_{GABA}= {g}_{GABA}*{s}_{GABA}*\left(V- {E}_{GABA}\right)$$

where, *V* represents the membrane voltage of the DA neuron, ${E}_{GABA}$ denotes the GABA reversal voltage and the GABA conductance, ${g}_{GABA}$, is the parameter quantifying the GABA current. The inhibitory input ${I}_{GABA}$ in Equation (1) is provided by a population of 30 GABAergic neurons, whose dynamics are described in Section "VTA GABAergic neuron model ()" (Equation (6)) and illustrated in Fig. 1.

### Control method

The main objective of the current study is to develop a robust controller capable of compensating the disturbances, perturbations, and uncertainties arising from the model’s structure. To this end, the robust ST-SMC is proposed and its performance is evaluated with DT, P, and PID controller methods. The controllers generate the control input (stimuli pulses to the DA neuron) based on two different outputs of the system, membrane voltage of the DA neuron or its firing rate. To implement the proposed ST-SMC, Equation (1) is considered with the first-order time-varying nonlinear system

7 $$\dot{x}=f\left(x\right)+g(x)u, x\in {\mathbb{R}},u\in {\mathbb{R}}$$

where, *x* represents the measured membrane voltage or the firing rate of the DA neuron and *u* is the control input (continuous DBS pulses). The nonlinear and unknown functions $f\left(x\right)$ and $g\left(x\right)$, characterize the system dynamics and regulate the impact of the control input on the system, respectively. The objective of the controller is to guarantee the convergence of the system’s state variable *x(t)* to the desired state variable ${x}_{d}(t)$, in the presence of system parameter uncertainties and external disturbances. Equation (7) should be interpreted as a control-oriented input-output representation of the regulated neuronal dynamics rather than as a full analytical reduction of the complete biophysical model introduced in Sections "VTA dopaminergic neuron model" and "VTA GABAergic neuron model ()". For membrane voltage regulation, the stimulation current enters directly into the membrane equation of the DA neuron, which yields a relative degree of one with respect to the regulated output. For firing rate regulation, the control action affects the firing rate through the membrane-voltage and spike-generation pathway, and the corresponding input-output dynamics are treated in the same control-oriented framework. In both cases, the internal ionic currents, gating-variable dynamics, and the inhibitory contribution of the GABAergic population are embedded in the lumped nonlinear term of the control-affine representation.

#### Assumption 1

The desired state variable ${x}_{d}(t)$ is a continuous function that is measurable.

The tracking error is defined by $e\left(t\right)=x\left(t\right)-{x}_{d}(t)$ and to design the robust ST-SMC, the sliding surface variable to reflect the desired behavior of the system is given by^32^

8 $$s\left(x,t\right)={(\frac{d}{dt}+\lambda )}^{n-1} e\left(t\right)$$

where in Equation (8), *λ* is a positive constant and *n* is the order of the system. Given the model’s relative degree of one (as indicated by Equation (7)), $s\left(x,t\right)=e(t)$.

#### Remark 1

Assumption 1 is introduced to ensure the well-posed definition of the tracking error and the sliding surface in the proposed control framework. In the first regulation approach considered in this study, where the membrane voltage of the VTA DA neuron is directly controlled, the desired signal ${x}_{d}(t)$ represents a time-varying voltage trajectory. Therefore, continuity and measurability of ${x}_{d}(t)$ are required to allow real-time computation of the tracking error $e\left(t\right)$ and the corresponding sliding surface defined in Equation (8). It is worth noting that Assumption 1 is not required for the second regulation approach, in which the control objective is defined in terms of the neuronal firing rate. In this case, the desired signal corresponds to a constant firing rate setpoint rather than a time-varying trajectory. As a result, the continuity requirement becomes trivial and the measurability condition is naturally satisfied, since only a fixed reference value is needed.

#### Remark 2

In the proposed formulation, the regulated output is selected as either the DA neuron membrane voltage or the DA neuron firing rate. Since the considered input-output dynamics have relative degree one with respect to the control input, the sliding variable in Equation (8) reduces to the tracking error itself. Therefore, the convergence analysis of the proposed ST-SMC is carried out on the sliding-variable dynamics associated with the regulated output, while the remaining internal neuronal and synaptic dynamics are incorporated into the lumped nonlinear term of the control-oriented model.

The proposed ST-SMC was utilized as the higher-order sliding mode controller (HOSMC) and represents an advancement over conventional SMCs by mitigating the chattering phenomenon and enhancing the performance of complex dynamic systems^33^. In contrast to classical SMC, which employs a single sliding surface, HOSMC utilizes higher-order derivatives of the sliding surface to achieve smoother control. These controllers $s\left(x,t\right)=0$ improve robustness in the presence of uncertainties and external disturbances. In classical SMC, the control law is designed to ensure that $s\left(x,t\right)=0$ in finite-time. Conversely, in SMC of order *n*, the objective is to drive the derivatives of the sliding surface to zero up to the (*n-1*)-th order in finite-time^34^ using a continuous, time-dependent control input $u\left(t\right)$

9 $$s=\dot{s}=\ddot{s}=\cdots ={s}^{\left(n-1\right)}=0$$

To explicitly connect the controller design to the exact models of Sections "VTA dopaminergic neuron model" and "VTA GABAergic neuron model ()", the derivative of the sliding variable is evaluated along the closed-loop neuronal dynamics. Since *s(x,t)=e(t)* for the considered relative-degree-one formulation, differentiating the tracking error yields a sliding-variable dynamics of the form

10 $$\dot{s}(t)=\phi (x,t)+g(x,t)u(t),$$

where, *ϕ (x,t)*collects the lumped nonlinear neuronal dynamics, including the intrinsic ionic currents of the DA neuron, the gating-state effects, the synaptic terms, and the contribution of the GABAergic population through ${I}_{\mathrm{G}\mathrm{A}\mathrm{B}\mathrm{A}}$, while *g(x,t)* denotes the effective input gain. Therefore, the exact biophysical model is preserved in the stability analysis through the induced sliding-variable dynamics. To achieve the finite-time convergence, the following conditions are assumed^32^:

$$0<{K}_{m}\le \left|\frac{\partial }{\partial u}\dot{s}(t,x,u)\right|\le {K}_{M}$$

11 $$\left|\frac{\partial }{\partial t}\dot{s}\left(t,x,u\right)+\frac{\partial }{\partial x}\dot{s}\left(t,x,u\right)f(t,x,u)\right|\le {C}_{0}$$

$$\left|s\right|\le {s}_{0}$$

where in Equation (11), the parameters ${K}_{m}$, ${K}_{M}$, ${C}_{0}$​, and ${s}_{0}$​ are positive constants. The proposed ST-SMC necessitates only the measurement of *s* and the controller does not need any prior knowledge of the unknown model dynamics in Equation (1) and ensures the robustness of the closed-loop system against parameter uncertainties and external disturbances^35^.

#### Remark 3.

The stability argument employed in this study follows the standard Lyapunov-based finite-time convergence framework of the super-twisting algorithm. In the present work, a new Lyapunov function is not constructed for the full biophysical state model of the DA neuron and the GABAergic population. Instead, once the exact neuronal dynamics are written in the control-oriented sliding form and the boundedness and non-vanishing gain conditions in Equation (10) are satisfied, the well-established Lyapunov-based super-twisting stability results can be invoked for the sliding-variable subsystem.

To design the control input, the continuous control law $u\left(t\right)$ comprises two continuous terms that are invariant with respect to the first-time derivative of the sliding variable ($s\left(x,t\right)$). The algorithm is defined by the control law^33^

12 $$u\left(t\right)=-\lambda {.\left|s\right|}^{\rho }.sign\left(s\right)+\varphi (t)$$

$$\dot{\varphi }\left(t\right)=-W.sign(s)$$

where in Equation (12), *W*, *λ*, and *ρ* are the controller parameters. The sufficient conditions for finite-time convergence to the sliding surface are as follows^33^:

$$W>\frac{{C}_{0}}{{K}_{m}}$$

13 $${\lambda }^{2}\ge \frac{{4C}_{0}}{{K}_{m}^{2}}\frac{{K}_{M}(W+{C}_{0})}{{K}_{m}(W-{C}_{0})}$$

$$0<\rho \le 0.5$$

The controller parameters are selected according to the admissible bounds imposed by the sliding dynamics. Consequently, when the controller parameters are selected to satisfy the sufficient inequalities in Equation (13), the standard super-twisting stability conditions are fulfilled for the resulting sliding-variable dynamics. Under these conditions, the derivative of the corresponding standard Lyapunov function for the super-twisting subsystem becomes negative along the closed-loop trajectories, which ensures finite-time convergence of the sliding variable to zero. Since *s(x,t)=e(t)* in the present formulation, this result implies finite-time convergence of the tracking error for both membrane voltage regulation and firing rate regulation. Additionally, the dynamic of the tracking error ($\dot{e}\left(t\right)=\dot{s}(x,t)$) converges to zeros in finite-time. It follows from the establishment of a second-order sliding mode and the super-twisting theorem, which guarantees finite-time convergence to {$s=0,\dot{s}=0$}.

#### Remark 4

To ensure a robust controller and achieve finite-time convergence, large values for the parameters *W* and *λ* are often required. However, utilizing large values for *W* and *λ* increases the amplitude and energy of the control signal which is a limiting factor in practical applications. Thus, the parameters λ and *W* are initially selected to ensure robustness against uncertainties and disturbances, and are subsequently adjusted in a stepwise manner to balance tracking accuracy, chattering reduction, and control energy^32,33^. This procedure allows the controller to achieve accurate tracking performance with minimal control effort, making the proposed ST-SMC particularly suitable for practical implementation, especially in firing rate–based regulation scenarios. The parameter ρ is selected within the interval (0, 0.5], with values closer to 0.5 reducing high-frequency switching effects.

#### Remark 5

Despite the nonlinear and uncertain nature of the considered VTA neuronal dynamics, the proposed ST-SMC framework involves a relatively small number of control parameters, namely λ, ρ, and *W*. This is a direct consequence of adopting a second-order sliding mode strategy that does not require explicit knowledge or online estimation of the unknown nonlinear functions $f\left(x\right)$ and $g\left(x\right)$ in (7). Unlike classical first-order sliding mode or adaptive SMC formulations, which typically necessitate additional estimators (e.g., fuzzy or neural approximators for estimation of dynamics $f\left(x\right)$ and $g\left(x\right)$) and consequently introduce a large number of tuning parameters, the super-twisting algorithm enables robust regulation using a compact and low-complexity control structure.

## Results

This section presents the simulation results obtained from the closed-loop control of the VTA neuronal model and provides a quantitative evaluation of controller performance. The evaluation criteria for the performance of the controllers are the root mean square error (RMSE) and the energy of control input. To evaluate the tracking accuracy, RMSE of the tracking is computed as follows:

14 $$RMSE=\sqrt{\frac{1}{T}\sum_{t=1}^{T}{e(t)}^{2}}$$

where, *T* is the number of samples in the total time of the simulation and *e(t)* is the tracking (membrane voltage or the firing rate) error for each sample. The energy of the control input is calculated as^36^.

15 $$Energy=\sqrt{\frac{1}{T}\sum_{t=1}^{T}{u(t)}^{2}}$$

**Fig. 2:**
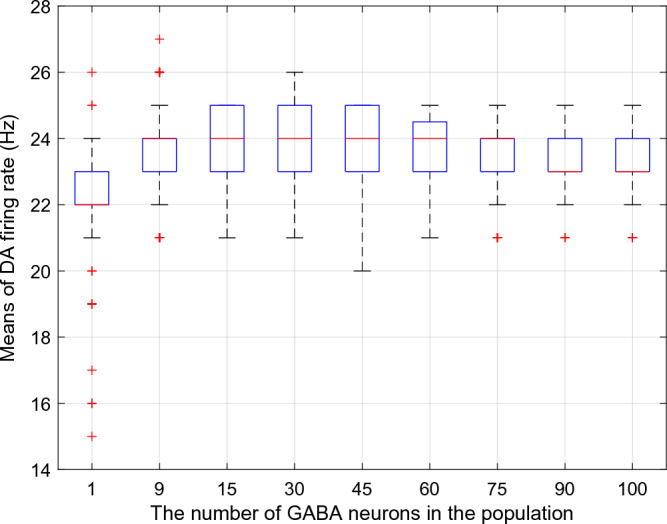
The box plots of the firing rate results of the DA neuron obtained from 100 simulation runs for each population, with each run having a duration of 1000 ms.

The schematic of the model and the closed-loop control system through two control algorithms, membrane voltage control and firing rate control of the dopamine neuron are indicated in Fig. 1. All simulations, including model and controllers implementation were performed in the Simulink environment of MATLAB R2020b, using the Ode4 (Runge-Kutta) solver on a system equipped with a CORE i7 processor, 32 GB DDR4 RAM, and an Intel (R) Core (TM) i7-9700K @ 3.60GHz CPU under Windows 10. The Simulink time step was selected to be 0.01 ms and the closed-loop system simulation repeated 10 times for every control algorithm and the total running time of each simulation was considered to be 10 s. To determine the number of neurons in the GABAergic population of the model, it is acknowledged that the precise count of GABAergic neurons transmitting signals to the DA neuron remains uncertain. Moreover, given the significant role of GABAergic neurons in modulating the activity of DA neurons, it is reasonable to expect that multiple GABAergic neurons may interact with a single DA neuron^30^. Therefore, a series of 100 simulations was performed for each GABAergic population to investigate their influence on the firing rate of a DA neuron model. The results of the firing rate (Hz) distribution for nine different populations are visualized using box plots in Fig. 2. The results show that the model demonstrates a relatively stable firing rate across a wide range of GABAergic neuron counts. Throughout the study in the simulation results of the model and the controllers, a population of 30 GABAergic neurons with the random initial conditions was considered to project onto a single DA neuron.

**Fig. 3:**
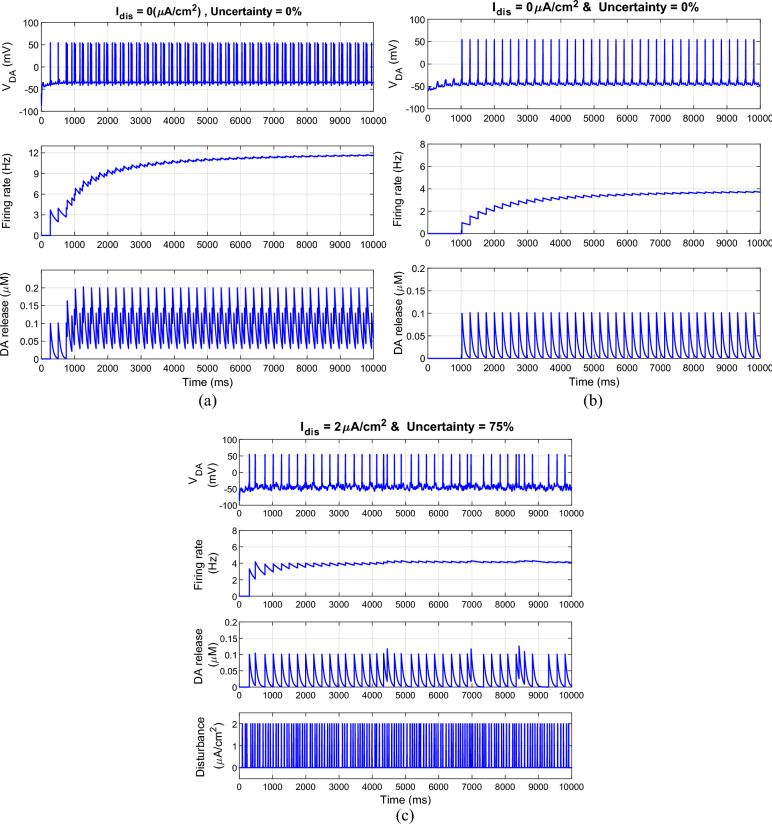
Typical results of the membrane voltage, firing rate, dopamine release for DA neuron model. (**a**) DA neuron firing rate of 10 Hz with no external disturbances and parameter uncertainties. (**b**) DA neuron firing rate of 4 Hz with no external disturbances and parameter uncertainties. (**c**) DA neuron firing rate of 4 Hz in the presence of external disturbances with an amplitude of 2 µA.cm^−2^ and 75% parameters uncertainty.

The simulation results of the DA neuron model are presented in Fig. 3. By regulating the four key model parameters ${I}_{0},{Mg}^{2+},{X}_{APA}$ and ${X}_{TTX}$ two different dopamine levels were simulated for the model in the results of Figs. 3(a) and (b), each affecting the firing rate of DA neurons with the values of 10 and 4 Hz, respectively. The allowable range for selecting these parameter values and their specific values are summarized in Table 1. To model the external disturbance, a non-regular constant current with a pulse amplitude of 2 µA.cm^−2^ and a pulse width of 1 ms was applied to the dynamic equation of the DA neuron in Equation (1). The pulse is generated based on a gamma distribution with a frequency of 14 Hz. Moreover, the conductance values of the ionic currents in DA neuron were varied by 75% around their nominal values during the entire time (10 s) of the simulation. The variation of each parameter was obtained by passing a uniformly distributed sequence, with appropriate selection of minimum and maximum values, through a fifth-order Butterworth filter with a natural frequency of 0.05 Hz, and then adding the constant nominal value of the parameter. The results of Fig. 3(c) incorporate the effects of external disturbances with an amplitude of 2 µA.cm^−2^ and 75% uncertainty for the parameters. The interesting observation is that by incorporating the effect of external disturbances and parameter uncertainties to the model, the behavior of the spiking in the membrane voltage of DA neuron model changes from a periodic state to a more realistic random one while the firing rate of the neuron remains unchagned in the value of about 4 Hz.

**Table 1:** Parameters that regulate the firing rate of the DA neuron model.

Parameter	Allowable range	Specific value
${I}_{0}$	[0, ∞]	0.5
${Mg}^{2+}$	[0, ∞]	0.75
${X}_{APA}$	[0, 1]	1
${X}_{TTx}$	[0, 1]	1

### Results of super-twisting sliding mode controller (ST-SMC)

In this section, the performance of the proposed ST-SMC is evaluated and compared with the P, DT, and PID controllers. The assessment is conducted based on quantitative performance criteria, including RMSE (Equation (14)), which provides a normalized measure of average tracking accuracy and the control input energy (Equation (15)), which reflects the required control effort. Together, these metrics enable a systematic evaluation of tracking accuracy, robustness, and control efficiency under external disturbances and parameter uncertainties. For the single-parameter controllers (P and DT), parameter tuning was performed through the grid search based on the RMSE and physiological constraints. Initially, relatively large gain values were considered to identify the upper operating range of each controller parameter. However, excessively large control gains produced unrealistically high injected currents into the DA neuron. Because the externally applied control current directly enters the membrane voltage dynamic together with the intrinsic ionic currents, an excessively strong control action can dominate the natural neuronal currents and distort the membrane dynamics. Under such conditions, the membrane voltage may enter saturation, causing the neuron to lose its normal spiking behavior and resulting in non-physiological responses. Therefore, the gain of each controller was progressively reduced until the minimum RMSE was achieved while preserving physiologically meaningful neuronal firing activity. For the PID controller, the parameters were determined using a greedy approach based on minimization of the tracking error (RMSE) under the same criterion applied to all controllers. The proportional gain was first selected, which corresponds to the gain used in the optimally tuned P controller. Subsequently, the integral and derivative gains were tuned sequentially. For both gains, the tuning process was initialized from relatively large values (*łe 1*), and the gains were then progressively reduced. It is worth noting that decreasing these gains led to a reduction in RMSE, and that the best performance was achieved as they approached zero with very small non-zero lower bounds. As a consequence, these lower bound values were selected as the smallest nonzero gains that preserve numerical distinction from the proportional controller while maintaining near-optimal performance.

#### DA neuron membrane voltage control

**Fig. 4:**
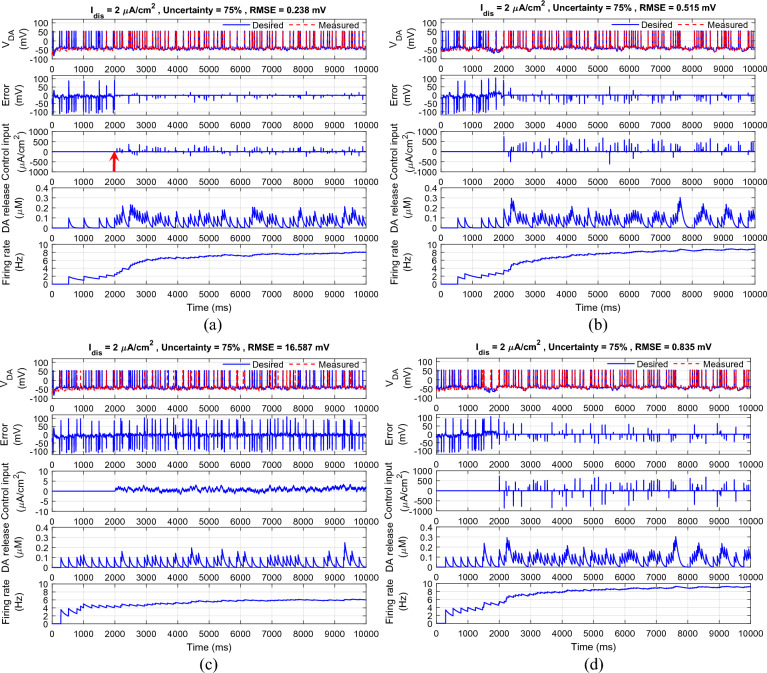
Typical tracking results of the controllers with 75% uncertainty in the nominal conductance values of the ionic currents of the DA neuron and the presence of external disturbance current. (**a**) ST-SMC. (**b**) P controller. (**c**) DT controller. (**d**) PID controller.

In this approach, the objective is to control the membrane voltage of the VTA DA neuron to track the desired membrane voltage pattern. The bursts and dense spikes behaviors in the pattern of the DA neuron membrane voltage release considerably more dopamine than scattered and periodic spiking behavior. Therefore, to achieve an appropriate amount of dopamine in the reward circuit, a suitable pattern of DA neuron membrane voltage in VTA can be considered as the desired signal for the controller. Figure 4 shows the tracking results of the membrane voltage for the proposed ST-SMC (Fig. 4(a)), P (Fig. 4(b)), DT (Fig. 4(c)), and PID (Fig. 4(d)) controllers. To evaluate the performance of the controllers, external disturbance current with an amplitude of 2 µA.cm^−2^ was added to the DA neuron in the presence of 75% uncertainty for the conductance values of the ionic currents in DA neuron. The initial conditions for all controllers were randomly selected and the control input was applied to the model at *t* = 2000 ms (red arrow in Fig. 4(a)). The tracking error is defined as $e\left(t\right)=V\left(t\right)-{V}_{d}(t)$ where $V\left(t\right)$ is the membrane voltage of DA neuron and ${V}_{d}(t)$ denotes the desired voltage. The results show that the ST-SMC achieves better performance (RMSE_ST-SMC_ = 0.238 mV) than the other three controllers in tracking the desired voltage pattern (RMSE_P_ = 0.515 mV, RMSE_DT_ = 16.587 mV, RMSE_PID_ = 0.835 mV). Notably, the DT controller failed to perform the tracking and exhibited a significantly higher RMSE. Although the P controller showed relatively acceptable performance, it still exhibited a higher RMSE and a higher range of control input amplitudes compared to the ST-SMC.

**Fig. 5:**
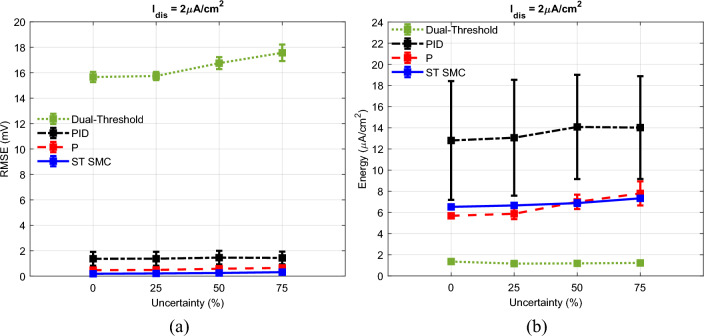
Mean results (± one standard deviation) obtained over 10 trials of the simulation for control of the DA neuron membrane voltage using proposed ST-SMC in comparison with the results of P, DT, and PID controllers in the presence of external disturbance current and different uncertainty. (**a**) RMSE. (**b**) Energy.

Fig. 5 shows the results of the mean RMSE (Fig. 5(a)) and mean control input energy (Fig. 5(b)) with ± one standard deviation obtained over 10 trials of the simulation for control of the DA neuron membrane voltage using proposed ST-SMC in comparison with the three P, DT, and PID controllers in the presence of external disturbance current and different uncertainty. The initial conditions of the model, the uncertainty of the conductances of the ionic currents, and the pattern of the external disturbance current applied to the model were randomly selected while being held constant across the three controllers. Each simulation lasted 10,000 ms and the control input was applied at *t* = 2000 ms. The results show that the DT controller exhibited poor performance, with mean RMSE greater than 14 mV, while the P controller and ST-SMC demonstrated well performance, with an average RMSE smaller than one mV. Furthermore, the ST-SMC indicates a lower mean RMSE in contrast to the P controller. Although the DT controller exhibits the lower mean control input energy, the higher uncertainty causes the larger standard deviation and mean RMSE.

#### DA neuron firing rate control

**Fig. 6:**
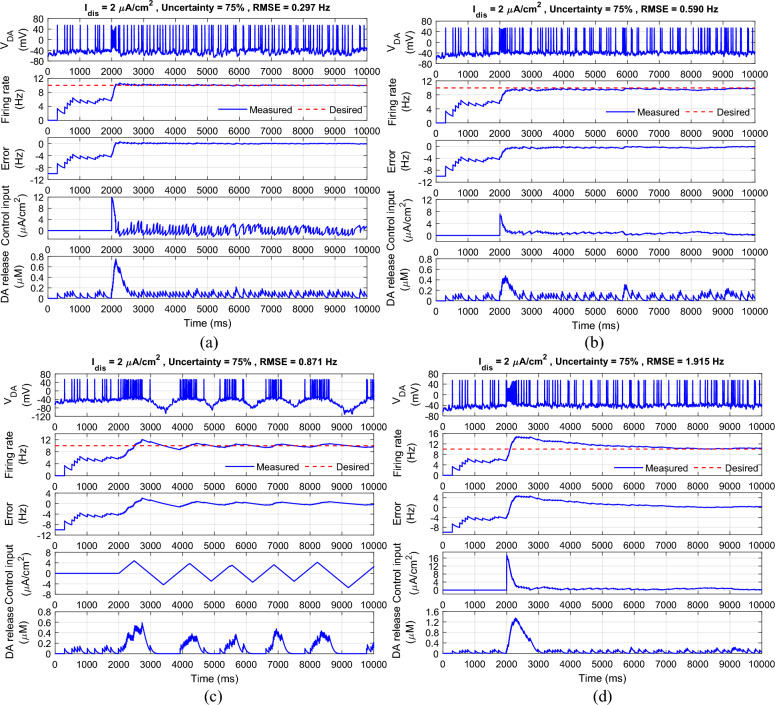
Typical results of DA neuron firing rate control with 75% uncertainty in the nominal conductance values of the ionic currents of the DA neuron and the presence of external disturbance current. (**a**) ST-SMC. (**b**) P controller. (**c**) DT controller. (**d**) PID controller.

**Fig. 7:**
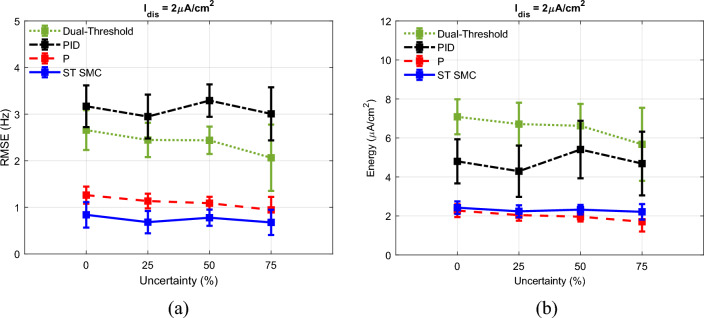
Mean results (± one standard deviation) obtained over 10 trials of the simulation for control of the DA neuron firing rate using proposed ST-SMC in comparison with the results of P, DT, and PID controllers in the presence of external disturbance current and different uncertainty. (**a**) RMSE. (**b**) Energy.

Given the important role of firing rate in determining the activity type and the amount of dopamine released by DA neurons, as well as the practical challenges of controlling DA neuron membrane voltage due to the complexity of its pattern, this section focuses on firing rate regulation as an alternative method to control the firing rate of DA neurons. In this approach, the model is initially set to a maximum firing rate of 4 Hz and the controller should force the DA neuron model to fire in the desired rate of 10 Hz (the maximum normal firing rate for DA neurons^37^). The tracking error in this approach is defined as $\mathrm{e}\left(\mathrm{t}\right)=\mathrm{F}\mathrm{R}\left(\mathrm{t}\right)-{\mathrm{F}\mathrm{R}}_{\mathrm{d}}$ where $\mathrm{F}\mathrm{R}\left(\mathrm{t}\right)$ is the measurable firing rate of DA neuron model and ${\mathrm{F}\mathrm{R}}_{\mathrm{d}}$ is the desired firing rate, set at 10 Hz. Fig. 6 presents the results of the three controllers in increasing the firing rate of the DA neuron to 10 Hz, under conditions where external disturbance current is applied to the DA neuron model, and 75% uncertainty is introduced to the model parameters. For all controllers, the control input is applied at *t* = 2000 ms. Fig. 6(a) corresponds to the performance of the ST-SMC controller with the RMSE of 0.297 Hz. The proposed controller (Fig. 6(a)) compensates the initial error by generating a large number of spikes immediately after the control input is applied (the amplitude of the control input peaks at approximately 12 µA.cm^−2^) and its response is faster in contrast with the P (Fig. 6(b)), DT (Fig. 6(c)), and PID (Fig. 6(d)) controllers. The results of Fig. 6(b) demonstrate that as the controller operates, a notable steady-state error is observed (RMSE_P_ = 0.590 Hz). Subsequently, the results of Fig. 6(c) illustrate that due to the firing rate exceeding the desired value, the DT controller decreases its amplitude (down to -4 µA.cm^−2^) to suppress all spikes until the firing rate returns to the desired level. The PID controller demonstrates less satisfactory performance compared to the ST-SMC, P, and DT controllers due to its immediate overshoot in firing rate and slow convergence to the desired value (RMSE_PID_ = 1.915 Hz). Figure 7 shows the mean results obtained over 10 trials of the simulation for control of the firing rate using proposed ST-SMC in comparison with the three controllers P, DT, and PID in the presence of external disturbance current and different uncertainty in terms of mean RMSE (Fig. 7(a)) and mean control input energy (Fig. 7(b)). The results of Fig. 7(a) indicate that the ST-SMC demonstrated better performance in comparison with the P, DT, and PID controllers across all levels of uncertainty. It can be observed that the mean control input energy of the ST-SMC and P controllers is nearly identical and lower than that of the DT and PID controllers.

To provide a quantitative comparison between the proposed ST-SMC and the benchmark controllers (P, DT, and PID) under external disturbance current with an amplitude of 2 µA.cm^−2^ in the presence of 75% uncertainty, the numerical performance indices were evaluated and summarized in Table 2. These indices include RMSE and energy of the control input, which collectively characterize tracking accuracy and control effort, respectively. For robustness and fairness, all results were obtained as the mean values over 10 independent simulation trials with different initial conditions. The results show that for the first control approach, where the membrane voltage of the VTA DA neuron is regulated, the proposed ST-SMC achieved a significantly lower tracking error compared to the P, DT, and PID controllers. Specifically, the mean RMSE of the ST-SMC was 0.31 mV, which is noticeably smaller than the corresponding values of 0.65, 17.57, and 1.43 mV obtained using the P, DT, and PID controllers, respectively. Despite this improved accuracy, the mean energy index of the control input required by the ST-SMC (7.33 µA.cm^−2^) remains lower than the energy index of the P and PID controllers, highlighting its efficiency in generating effective stimulation currents. Note that, although the control energy of the DT is lower than that of the proposed controller, it leads to poor tracking performance in terms of the RMSE. For the second control approach, based on firing rate regulation, similar performance improvements were observed. The ST-SMC successfully regulated the firing rate to the desired value with a mean RMSE of 0.67 Hz, outperforming the P (0.94 Hz), DT (2.06 Hz), and PID (3.00 Hz) controllers. Although the mean control energy of the ST-SMC (2.21 µA.cm^−2^) is slightly higher than that of the P controller, it achieves a lower RMS firing rate error. This indicates that the proposed method attains improved regulation accuracy with only a marginal increase in stimulation effort, reflecting an effective trade-off between precision and energy consumption.

**Table 2:** Mean performance indices (± standard error) over 10 independent simulation trials with different initial conditions for membrane voltage–based and firing rate–based control strategies under external disturbance current with an amplitude of 2 µA.cm^−2^ in the presence of 75% uncertainty.

	DA neuron membrane voltage control		DA neuron firing rate control	
Control method	RMSE (mV)	Energy (µA.cm^−2^)	RMSE (Hz)	Energy (µA.cm^−2^)
ST-SMC (proposed)	0.31 ± 0.05	7.33 ± 0.22	0.67 ± 0.28	2.21 ± 0.42
P	0.65 ± 0.09	7.79 ± 1.15	0.94 ± 0.28	1.70 ± 0.52
DT	17.57 ± 0.66	1.22 ± 0.25	2.06 ± 0.72	5.67 ± 1.93
PID	1.43 ± 0.5	14.01 ± 4.89	3.00 ± 0.60	4.68 ± 1.68

## Discussion

Considering the critical role of VTA dopaminergic signaling in emotion regulation, learning, memory, motivation, and reward-related behaviors, the ability to regulate the activity of VTA DA neurons is essential. In this paper, a robust control strategy based on the ST-SMC was employed to regulate dopamine release from DA neurons in VTA. The conductance-based dynamic model of the membrane voltage for the DA neuron, as described in Equation (1), comprises ionic and synaptic currents, with the control input added to the model as an external current. The performance of the proposed controller is compared with DT, P, and PID controllers to evaluate its effectiveness and robustness, based on RMSE and control input energy indexes. DT and P controllers have been utilized in closed-loop control of pathological beta oscillations in Parkinson’s disease, and the results have been extensively studied and benchmarked^38^. Additionally, several recent and advanced feedback controllers have been proposed for nonlinear systems, including softsign fractional-order controllers, sigmoid PID controllers, brain emotional learning-based intelligent controller (BELBIC) and cerebellar model articulation controller (CMAC)-based PID frameworks, as well as neuroendocrine PID controllers^21,39–43^. While these approaches have demonstrated strong performance in various applications, their design typically relies on nonlinear mappings, adaptive gain mechanisms, learning structures, or heuristic biologically-inspired parameters that implicitly depend on system characteristics and often require problem-specific tuning rules, iterative optimization, or training data^21,39–43^. In contrast, the proposed ST-SMC does not require precise knowledge of the system dynamics for implementation and can operate effectively in the presence of uncertainties and disturbances under standard sliding mode control assumptions^34,44^. Furthermore, ST-SMC is specifically designed to maintain stability and tracking performance without relying on learning-based adaptation or online retuning mechanisms, whereas the performance of other advanced controllers may degrade when operating conditions differ from those used during tuning or training, or when heuristic parameters are not optimally selected for the specific plant^21,39–43^. In terms of parameterization and tuning complexity, advanced controllers^39,42,43^ generally involve multiple adjustable parameters, including fractional orders, nonlinear shaping coefficients, learning rates, or memory-related weights, which may require iterative tuning, optimization algorithms, or training procedures. In comparison, the ST-SMC structure employs a comparatively smaller set of controller gains and does not inherently rely on iterative optimization or training procedures for operation ^34,44^. Additionally, learning-based controllers such as CMAC- and BELBIC-based approaches rely on adaptation laws, reward/sensory signal design, or memory-based learning mechanisms, which increase implementation complexity and computational overhead^39,43^. Similarly, several bio-inspired PID approaches, including neuroendocrine-based frameworks^40,41^ commonly utilize optimization or adaptation procedures for parameter adjustment, while ST-SMC is a non-learning-based strategy that does not require training datasets or data-driven calibration procedures^44^. Compared to the classical SMC, the ST-SMC significantly reduces chattering, which in turn lowers mechanical wear on actuators, decreases energy consumption, and minimizes sensor noise. These improvements collectively enhance its feasibility for practical applications. Given the inherent complexity of brain structure, the dynamic nature of the reward circuitry, and the limited understanding of precise neuronal interconnections including unmodeled external ionic currents, the parameters of the proposed model, such as ionic conductances and neuronal firing thresholds, exhibit intrinsic uncertainty. In nonlinear neural systems such as the VTA dopaminergic circuitry, unknown dynamics are often addressed by incorporating fuzzy, neuro-fuzzy, or adaptive estimators within sliding mode control frameworks. Although such approaches can approximate system nonlinearities, they typically introduce significant computational complexity and additional tuning parameters. In the present study, the ST-SMC framework is intentionally adopted to avoid explicit estimation of unknown dynamics, allowing robust regulation to be achieved using only measurable outputs. This choice reflects a design trade-off favoring computational efficiency and practical feasibility over model-dependent estimation accuracy. The ST-SMC has been selected as a robust control strategy to regulate the behavior of the model in the presence of uncertainties and external disturbances (results of Figs. 5 and 7). In the previous work^45^, a multi-input multi-output adaptive fuzzy terminal sliding mode controller (AFTSMC), combined with a fuzzy logic system (FLS) estimating the unknown nonlinear dynamics is employed to regulate the thalamic neuron populations in the basal ganglia–thalamic network for adaptive deep brain stimulation in Parkinson’s disease. In another previous study^46^, an analogous AFTSMC with FLS was applied to eliminate the oscillatory spiking behavior in a childhood absence epilepsy (CAE) dynamical model consisting of cortical, thalamic relay, and reticular nuclei neurons. In the aforementioned previous studies, the dynamics of the plant were directly incorporated into the controller structure, necessitating the use of an adaptive fuzzy approach to estimate these dynamics. This requirement led to a complex controller design, involving numerous mathematical equations and many control parameters, which had to be optimized through trial and error. In practical applications, however, controllers need to be as simple as possible to allow feasible hardware implementation. Consequently, the high complexity of the control structures in these previous studies poses significant challenges for practical implementation. In contrast, the proposed ST-SMC controller, despite its simple structure and without requiring the plant dynamics, exhibits high robustness in the presence of external disturbances which is suitable for practical implementation, particularly in systems subject to modeling uncertainties and external disturbances. Furthermore, a notable advantage of the proposed ST-SMC is its inherent suitability for hardware realization on platforms such as microcontrollers, FPGAs, and PLCs. The feasibility of such implementations has been demonstrated in various applications, including minimally invasive surgery using higher-order sliding mode observers^47^, real-time control of upper limb wearable robotic systems^48^, and closed-loop control of stimulation pulses in noninvasive deep brain stimulation, highlighting its robustness and adaptability in practical control systems.

In the current study, closed-loop control of the developed model was carried out using two distinct methods, control of the DA neuron membrane voltage and firing rate. In the first approach, the membrane voltage of a single VTA DA neuron was chosen as the desired signal, and its tracking was performed. The performance of the ST-SMC controller was evaluated under 75% parameter uncertainty and an external disturbance current of 2 μA.cm^−2^, and the result was compared with the three experimental DT, P, and PID controllers (Fig. 4). The results demonstrated that, from *t* = 2000 ms, when the control input was applied to the plant, the tracking of the desired signal was successfully achieved. This procedure was repeated for different desired signal patterns, and the results confirmed that the ST-SMC controller reliably tracked all desired signal patterns. However, in practical applications, the membrane voltage of a single neuron as a desired signal is not feasible, as it varies over time and across different subjects. Moreover, stable intracellular recording of action potentials requires periodic calibration of the experimental setup limiting the long-term applicability of this approach. To address these limitations and make the controller suitable for clinical applications, in the literature, relevant features including synchrony of brain neural oscillations and sub-band power signals which are both associated with disease symptoms were extracted from the recorded signals to control the output. In the current study, in the second approach, a fixed value of firing rate in 10 Hz was chosen as the desired control signal. During the early stage of applying the control input (2000–2500 ms) in Fig. 6, the ST-SMC exerts considerable effort, generating rapid successive spikes to compensate for the firing rate deficit. Within a short time interval, the firing rate rapidly increased, reaching the desired value of 10 Hz, and was subsequently maintained at this level (Fig. 6(a)). The simplicity of this control approach, combined with the use of firing rate (a feature far easier to measure than a single neuron’s membrane voltage) enhances its applicability for experimental and clinical implementation. Importantly, the firing rate of a single neuron can be derived from local field potential (LFP) signals with a highly accurate estimation without direct measurement of action potentials^49^. This makes the approach practical and feasible for real-world applications.

This study adopted a simplified approach to modeling the reward circuit, avoiding full biological detail to reduce complexity and minimize mathematical equations. The model focuses on the neurons of the VTA as the primary nucleus for dopamine production, representing their dynamics explicitly, while the activity of other nuclei was incorporated as disturbance currents. From a computational perspective, the proposed ST-SMC controller involves a fixed and limited number of algebraic operations at each sampling instant, including error computation, sliding surface evaluation, and control signal generation. This characteristic stands in contrast to adaptive fuzzy, neuro-fuzzy, or learning-based control frameworks^46,50^, which require online parameter estimation and iterative update laws, substantially increasing computational load and implementation complexity. The absence of model identification, adaptive estimators, or optimization routines makes the proposed control scheme well suited for real-time implementation on embedded platforms such as microcontrollers or FPGA-based systems, particularly in closed-loop neuromodulation applications where computational resources and power consumption are constrained. Nevertheless, some practical limitations remain. In particular, although firing rate–based regulation significantly improves feasibility compared to direct membrane voltage control, reliable estimation of firing rate in experimental settings still requires adequate signal acquisition and processing capabilities. In addition, delays and noise in neural recordings, as well as constraints on stimulation waveform shape and amplitude imposed by safety considerations, may influence closed-loop performance in real-world implementations. Addressing these aspects constitutes an important direction for future experimental and translational studies. Moreover, from a simulation point of view, for future studies, the model could be expanded to incorporate additional nuclei of the reward circuit, including the mPFC, which contributes to decision-making, executive control, and emotional regulation, and the NAc, which is central to reward processing and motivational behaviors^51^.

## Conclusion

In this study, a robust closed-loop control framework based on ST-SMC was developed for regulating dopamine-related dynamics in a microscopic VTA DA neuron model. Unlike conventional open-loop or purely descriptive approaches, the proposed method adopts a control-oriented formulation explicitly designed to handle nonlinearities, parameter uncertainties, and external disturbances. The controller was implemented at two complementary regulation levels: direct membrane voltage control and firing rate–based control. Under external disturbance current (2 µA.cm^−2^) and up to 75% parameter uncertainty, the proposed ST-SMC achieved a mean RMSE of 0.31 mV for membrane voltage regulation, significantly outperforming the P (0.65 mV), DT (17.57 mV), and PID (1.43 mV) controllers. For firing rate regulation, the proposed method achieved a mean RMSE of 0.67 Hz, compared to 0.94 Hz, 2.06 Hz, and 3.00 Hz for the P, DT, and PID controllers, respectively. Although the control energy required by the ST-SMC in the firing rate scenario was slightly higher than that of the P controller, the substantial reduction in tracking error demonstrates a favorable accuracy–energy trade-off. Beyond improved performance indices, the proposed framework benefits from a compact control structure that does not require explicit estimation of unknown nonlinear dynamics, thereby reducing computational complexity and facilitating real-time implementation in neuromodulation systems. Overall, the results confirm that the ST-SMC provides a robust, accurate, and practically feasible solution for closed-loop regulation of dopaminergic activity, offering a promising foundation for future experimental validation and translational applications.

## Supplementary Information

Supplementary Information.

## Data Availability

All data generated or analysed during this study are included in this published article.
