# Supplementary material for: Super-twisting sliding mode control of dopamine release in VTA dopaminergic neuron model: a simulation study
Source: Sci Rep. 2026 May 28;16:24429. doi: 10.1038/s41598-026-54776-7 (PMC13448069; doi:10.1038/s41598-026-54776-7)
Supplement: Supplementary file 1 [file 41598_2026_54776_MOESM1_ESM.docx]

**Supplementary Materials for “Super-twisting sliding mode control of dopamine release in VTA dopaminergic neuron model: a simulation study”**

Najme Soheilipour ^1^, Amir Akhavan ^1^, Ehsan Rouhani ^1^, Raha Rahimi ^1^

^1^ *Department of Electrical and Computer Engineering, Isfahan University of Technology, Isfahan 84156-83111, Iran.*

**Appendix A. Details of the ionic and synaptic currents of the DA neuron model**

This Appendix provides a comprehensive detail of the intrinsic and synaptic currents of the DA neuron, including the dynamic equations and relevant parameters. The currents can be classified into three categories: Spike generating and voltage-dependent ($I_{Na}$, $I_{K}^{DR}$, $I_{K}$, $I_{Na}^{pers}$, and $I_{leak}$), calcium and calcium-dependent ($I_{Ca}^{L}$, and $I_{K}^{SK}$), and synaptic (AMPA, NMDA, and GABA) currents. The rapid processes involved in spike generation are governed by sodium dynamics, which are modeled using the Morris–Lecar method as follows:

| (S.1) | $I_{Na}= \bar{g}_{Na}m_{\infty}^{3}\left( V \right)h\left[ V-E_{Na} \right]$ |
| --- | --- |

where, $m_{\infty}$ and $h$ are the fast activation and slow inactivation. $\bar{g}_{Na}=150$ is the maximum conductance and $E_{Na}=55 \mathrm{mV}$ is a reversal voltage. The fast activation is described by:

| (S.2) | $m_{\infty}\left( V \right)= \frac{1}{2}\left[ 1-\tanh\left( \frac{p_{2}-V}{p_{3}} \right) \right]$ |
| --- | --- |

In equation (S.2), $p_{2}$ is set to $-14 \mathrm{mV}$ and $p_{3}$ is 11.9. The gating variable $h$ is obtained with:

| (S.3) | $\frac{dh}{dt}=\alpha_{h}\left( V \right)\left( 1-h \right)-\beta_{h}\left( V \right)h$ |
| --- | --- |

where

| (S.4) | $\alpha_{h}\left( V \right)= \frac{h_{a1}}{2}\left[ 1+\tanh\left( \frac{h_{a2}-V}{h_{a3}} \right) \right]$  $\beta_{h}\left( V \right)=\frac{h_{b1}}{2}\left[ 1-\tanh\left( \frac{h_{b2}-V}{h_{b3}} \right) \right]$ |
| --- | --- |

In Equation )S.4(, the parameters are assigned the following values: $h_{a1}=0.05 ms^{-1}, h_{a2}=-42 mV, h_{a3}=15, h_{b1}=1.10 ms^{-1}, h_{b2}=-10 mV$ and $h_{b3}=8.5$. Furthermore, the delayed rectifier potassium current is characterized as follows:

| (S.5) | $I_{K}^{DR}\left( V \right)= \bar{g}_{K}^{DR}n^{4}\left[ V-E_{K} \right]$ |
| --- | --- |

Where, $\bar{g}_{K}^{DR}=4$ and the reversal voltage $E_{K}=-90 \mathrm{mV}$. The gating variable $n$ is denoted with the following equation:

| (S.6) | $\frac{dn}{dt}=\alpha_{n}\left( V \right)\left( 1-n \right)-\beta_{n}\left( V \right)n$ |
| --- | --- |

where,

| (S.7) | $\alpha_{n}\left( V \right)= \frac{n_{a1}}{2}\left[ 1-\tanh\left( \frac{n_{a2}-V}{n_{a3}} \right) \right]$  $\beta_{n}\left( V \right)=\frac{n_{b1}}{2}\left[ 1+\tanh\left( \frac{n_{b2}-V}{n_{b3}} \right) \right]$ |
| --- | --- |

In Equation )S.7(, the parameters are: $n_{a1}=1 ms^{-1}, n_{a2}=100 mV, n_{a3}=80, n_{b1}=2 ms^{-1}, n_{b2}=-30 mV$ and $n_{b3}=10$. Furthermore, the generic potassium current, $I_{K}$, which encompasses the full range of potassium currents, is described with

| (S.8) | $I_{K}\left( V \right)=\bar{g}_{K}\left( \frac{1}{1+exp\frac{-\left( V-k_{2} \right)}{k_{3}}} \right)\left( V-E_{K} \right)$ |
| --- | --- |

where, $\bar{g}_{K}=0.4$, $k_{2}=-15 \mathrm{mV}$ and $k_{3}$ is $7 \mathrm{mV}$ . $I_{Na}^{pers}$ is represented as follows:

| (S.9) | $I_{Na}^{pers}\left( V \right)=g_{pers}\frac{1.1}{1+e\frac{\left( -50-V \right)}{3}}\left( V-E_{Na} \right)$ |
| --- | --- |

where, $g_{pers}=0.002$. The leakage current is modeled by

| (S.10) | $I_{leak}=g_{L}\left( V-E_{leak} \right)$ |
| --- | --- |

with a conductance of $g_{L}=0.015$ and a reversal voltage of $E_{leak}=-50 \mathrm{mV}$.

The L-type calcium current is modeled according to Equation (S.11), with $E_{Ca}=100 \mathrm{mV}$ and $G_{Ca}^{L}\left( V \right)$ is specified in Equation (S.12).

| (S.11) | $I_{Ca}^{L}\left( V \right)=G_{Ca}^{L}\left( V \right)\left( V-E_{Ca} \right)$ |
| --- | --- |

| (S.12) | $G_{Ca}^{L}\left( V \right)=\bar{g}_{Ca}^{L}\left( \frac{\alpha_{C}\left( V \right)}{\alpha_{C}\left( V \right)+\beta_{C}\left( V \right)} \right)^{4}$  $\alpha_{C}\left( V \right)=-0.0032\left( \frac{V+50}{\exp\left( \frac{-\left( V+50 \right)}{5} \right)-1} \right)$  $\beta_{C}\left( V \right)=\exp\left( \frac{-\left( V+55 \right)}{40} \right)$ |
| --- | --- |

In Equation )S.12(, $\bar{g}_{Ca}^{L}$ is set to $0.8{ms}/{{cm}^{2}}$. The calcium-dependent SK potassium current is represented by

| (S.13) | $I_{K}^{SK}\left( V.u \right)=G_{SK}\left( u \right)\left( {V-E}_{K} \right)$ |
| --- | --- |

where,

| (S.14) | $G_{SK}\left( u \right)=\bar{g}_{SK}\left( \frac{u^{4}}{u^{4}+K_{1}^{4}} \right)$ |
| --- | --- |

In Equation )S.14), $\bar{g}_{SK}=2{ms}/{{cm}^{2}}$ and $K_{1}=125.8 nM$. Calcium primarily enters the cell through L-type calcium channels and is extruded via the calcium pump. Accordingly, the concentration of calcium ($u$), is denoted with

| (S.15) | $\frac{du}{dt}=\frac{{2f}_{Ca}}{r}\left( \frac{I_{Ca}^{L}}{H}-I_{pump} \right)$ |
| --- | --- |

where, the cytosolic buffering constant is $f_{Ca}=0.01$. The term $H$ represents a mass term incorporating both the calcium capacity and the Faraday constant ($H\approx0.0193$). The neuron's radius $r=20 \mu m$, and the dynamics of the pump are described by

| (S.16) | $I_{pump}\left( u \right)= \frac{M_{pump}u}{(u+K_{pump})}$ |
| --- | --- |

where, in equation (S.16) $M_{pump}=500 nM\mu m/ms$ and $K_{pump}=500 nM$.

Dopaminergic cells receive excitatory glutamatergic input mediated by AMPA and NMDA receptors. The AMPA current is modeled with

| (S.17) | $I_{AMPA}= g_{AMPA}\left( V-E_{AMPA} \right)$ |
| --- | --- |

where, $E_{AMPA}=0 \mathrm{mV}, g_{AMPA}=0.002$. The NMDA current, a key modulator of dopamine dynamics, is modeled according to the following Equation:

| (S.18) | $I_{NMDA}= G_{NMDA}\left( V \right)\left[ V- E_{NMDA} \right]$ |
| --- | --- |

where, $E_{NMDA}=0 \mathrm{mV}$ and

| (S.19) | $G_{NMDA}\left( V \right)= \left( \frac{\bar{g}_{NMDA}^{stim}+\bar{g}_{NMDA}^{c}}{1+0.28\left[ Mg \right]e^{-m_{e}*\left( V+20 \right)}} \right)$ |
| --- | --- |

In Equation (S.19), $Mg=0.5 \mu M$ denotes the magnesium concentration and $g_{NMDA}^{-stim}=0.1$, $g_{NMDA}^{-c}=0.01$, and $m_{e}=0.08$. Equation (S.20) describes the simplified model of the GABA current, neglecting the number of neurons producing the current. In this model, $g_{GABA}=0.15{ms}/{{cm}^{2}}$ represents the conductance, and $E_{GABA}=-65 \mathrm{mV}$ is the reversal voltage.

| (S.20) | $I_{GABA}= g_{GABA}\left( V- E_{GABA} \right)$ |
| --- | --- |

**Appendix B. Details of the ionic currents of the GABAergic neuron model**

This appendix presents a model for a single GABA neuron. The membrane voltage dynamics of the neuron are determined by the sum of its ionic and synaptic currents. The ionic current $I_{Nag}$ is described as follows:

| (S.21) | $I_{Nag}= \bar{g}_{Nag}m_{\infty}^{3}\left( v_{i} \right)h\left( E_{Na}-v_{i} \right)$ |
| --- | --- |

where, $\bar{g}_{Nag}=22{ms}/{{cm}^{2}}$ and $E_{Na}=55 \mathrm{mV}$. The activation variable $m_{\infty}$ is determined by

| (S.22) | $m_{\infty}(v_{i})=\frac{\alpha_{m}}{\alpha_{m}+\beta_{m}}$ |
| --- | --- |

where,

| (S.23) | $\alpha_{m}=\frac{0.1\left( v_{i}+30 \right)}{1-\exp\left( \frac{-\left( v_{i}+30 \right)}{10} \right)}$  $\beta_{m}=4exp(\frac{-(v_{i}+55)}{18})$ |
| --- | --- |

The inactivation variable $h$ is governed by

| (S.24) | $\frac{dh}{dt}=\alpha_{h}\left( 1-h \right)-\beta_{h}h$ |
| --- | --- |

where,

| (S.25) | $\alpha_{h}=0.07\exp\left( \frac{-\left( v_{i}+53 \right)}{20} \right)$  $\beta_{h}=\frac{1}{1+exp(\frac{-(v_{i}+23)}{10})}$ |
| --- | --- |

$I_{Kg}$ current is denoted with the following equation:

| (S.26) | $I_{Kg}= \bar{g}_{Kg}n \left( E_{K}-v_{i} \right)$ |
| --- | --- |

where, $\bar{g}_{Kg}=7{ms}/{{cm}^{2}}$ and $E_{K}=-90 \mathrm{mV}$. The activation variable $n$ is governed by

| (S.27) | $\frac{dn}{dt}=\alpha_{n}\left( 1-n \right)-\beta_{n}n$ |
| --- | --- |

where,

| (S.28) | $\alpha_{n}=\frac{0.01\left( v_{i}+29 \right)}{1-\exp\left( \frac{-\left( v_{i}+29 \right)}{10} \right)}$  $\beta_{n}=0.0875exp(\frac{-(v_{i}+39)}{80})$ |
| --- | --- |

The leakage current $I_{lg}$ is defined as

| (S.29) | $I_{Kg}= \bar{g}_{Kg}n \left( E_{K}-v_{i} \right)$ |
| --- | --- |
| (S.30) | $I_{lg}= g_{lg}\left( E_{lg}-v_{i} \right)$ |

where, $g_{lg}=0.05{ms}/{{cm}^{2}}$ and $E_{lg}=-51 \mathrm{mV}$.
